# The Role of ATRX in Glioma Biology

**DOI:** 10.3389/fonc.2017.00236

**Published:** 2017-09-29

**Authors:** Pravanya Nandakumar, Alireza Mansouri, Sunit Das

**Affiliations:** ^1^Division of Neurosurgery, St. Michael’s Hospital, University of Toronto, Toronto, ON, Canada; ^2^Center for Cancer Research, Neuro-Oncology Branch, National Cancer Institute, National Institutes of Health, Bethesda, MD, United States; ^3^Division of Neuro-Oncology, Johns Hopkins University, Baltimore, MD, United States; ^4^The Arthur and Sonia Labatt Brain Tumour Centre, Hospital for Sick Kids, University of Toronto, Toronto, ON, Canada

**Keywords:** glioma, oligodendroglioma, astrocytoma, IDH, ATRX

## Abstract

The current World Health Organization classification of CNS tumors has made a tremendous leap from past editions by incorporating molecular criteria in addition to the pre-existing histological parameters. The revised version has had a particular impact on the classification of diffuse low-grade gliomas and their high-grade variants. The ATRX status is one of the critical markers that define the molecular classification of gliomas. In this review, we will first provide an overview of the role of ATRX in regular cell biology. Furthermore, the role of ATRX in tumorigenesis, specifically gliomas, is comprehensively elucidated. The possible correlation of ATRX status with other genetic/epigenetic modifications is also presented. We conclude by discussing some of the challenges associated with incorporating ATRX status assessment into routine clinical practice while also exploring opportunities for future diagnostics/therapeutics in gliomas based on ATRX status.

## The World Health Organization (WHO) Classification of CNS Tumors

The latest version of the WHO classification scheme of the central nervous system tumors has undergone an iterative process of transformation since its inception in 1979 (1), reflecting the emergence of the novel innovative technology and clinical findings of a particular era. Initially based on pure histological features (2, 3), immunohistochemical features were incorporated into the armamentarium of diagnosis in 1993 (2, 4). The year 2000 saw the integration of genetic profiles of tumors, in addition to immunohistochemical information; furthermore, salient features of the epidemiology of each tumor subgroup were also incorporated (5). The grouping of tumors based on histological phenotype was initiated in the 2007 version, wherein diffuse gliomas were subcategorized as astrocytomas, oligodendrogliomas, or oligoastrocytomas and each subcategory was further recognized by its grade of malignancy (2). Although at the time, characteristic genetic alterations within histological categories were starting to emerge, it was not until 2016 that they were implemented into the classification scheme. The current WHO classification of CNS tumors released in 2016 is a revised version of the 4th edition rather than a 5th edition (1) (Table 1). It has made a tremendous leap from past editions by incorporating molecular criteria in addition to the pre-existing histological parameters (1). One of the most important contributions of incorporating molecular features is envisioned to be the minimization of interobserver variability and, thus, striving toward improving diagnostic reproducibility (6). This has enabled a more robust approach toward diagnosis, prognostication, and management of CNS tumors (1).

**Table 1:** 2007 WHO classification scheme of diffuse glioma according to histology and grade.

Phenotype	Subtype	Grade
Astrocytic tumors	Pilocytic astrocytoma	I
	Diffuse astrocytoma	II
	Anaplastic astrocytoma	III
	Glioblastoma	IV

Oligodendroglial tumors	Oligodendroglioma	II
	Anaplastic oligodendroglioma	III

Oligoastrocytic tumors	Oligoastrocytoma	II
	Anaplastic oligoastrocytoma	III

**Figure 1:**
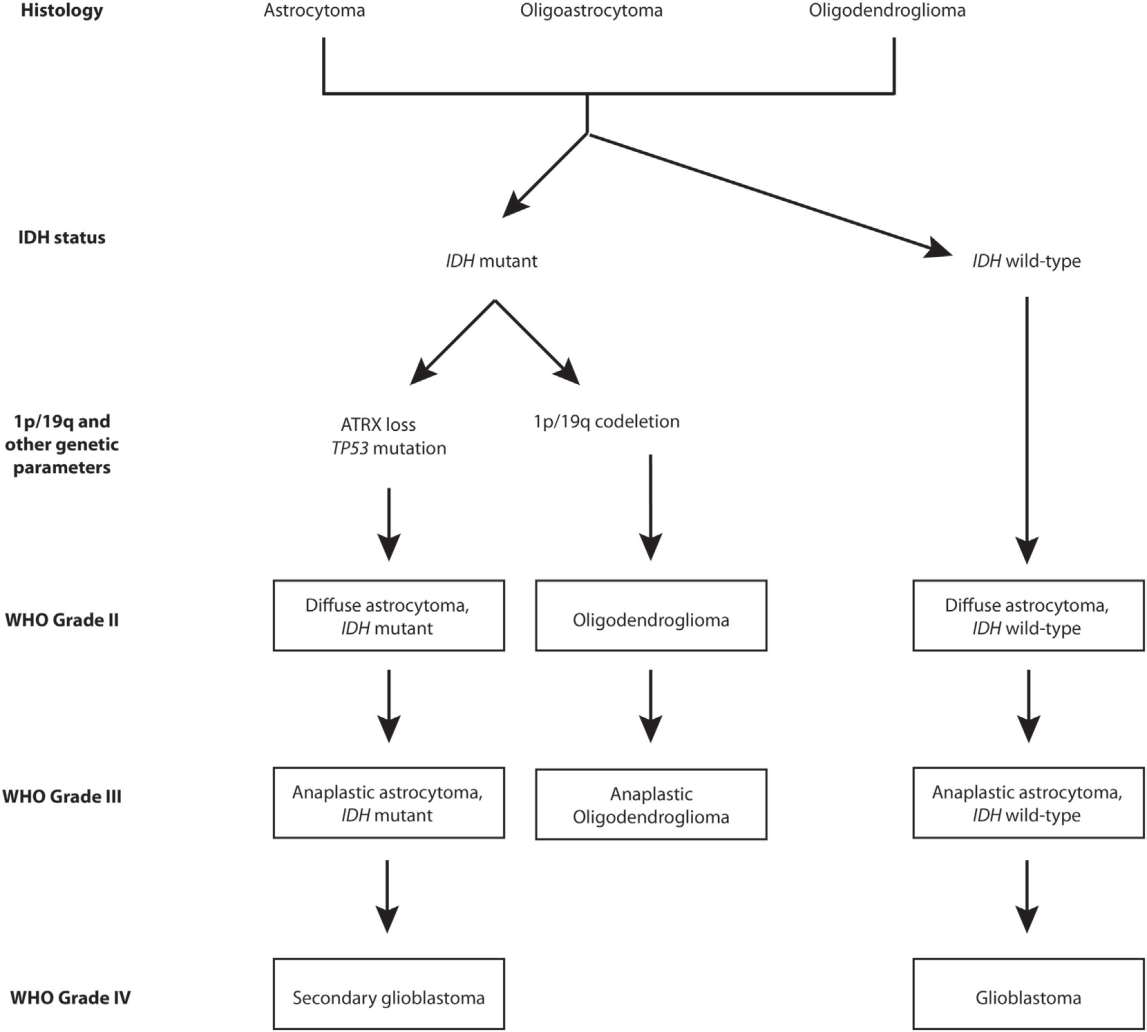
Schema of molecular alterations in various grades of gliomas.

The revised version has had a particular impact on the classification of diffuse low-grade gliomas and their high-grade variants (7). IDH mutation and 1p/19q codeletion statuses have captured the biological characteristics (DNA methylation, mRNA, DNA copy number, and microRNA) of lower-grade gliomas (LGGs) with a greater reliability than histological classes (8). In this regard, the four molecular parameters utilized for diffuse gliomas are absence/presence of IDH mutations, 1p/19q chromosomes codeletion, TP53 mutation, and ATRX loss (1). Figure 1 provides a graphical overview of the phylogeny of gliomas within the low- to high-grade spectrum. As shown in Figure 1, astrocytomas are either IDH mutants with ATRX loss and TP53 mutations or are IDH wild-type, whereas oligodendrogliomas are IDH mutants with 1p/19q codeletion. The diagnosis and management of diffuse low-grade gliomas based on these subcategories is a step toward the emergence of precision medicine. Therefore, it is critical for specialists involved in the field of neuro-oncology to have a thorough understanding of the particular molecular/genetic components of interest. To this end, detailed knowledge about the role of each component within regular cell metabolism and tumorigenesis is paramount. Furthermore, a fundamental understanding can outline avenues of therapeutic potential and future possibilities in the management of these complex tumors. In the following review, we will focus on the central role of the ATRX gene and its product in regular cell biology while also elucidating its role in tumorigenesis. Furthermore, we will highlight its potential role in clinical practice, aside from mere diagnostics. We conclude by addressing some of the challenges associated with the incorporation of such molecular markers in the future care of patients with gliomas.

## ATRX in Normal Biology

### Chromatin Remodeling

The ATRX gene was first discovered through a study assessing patients with the x-linked mental retardation (MR) syndrome (ATRX syndrome) presenting with α-thalassemia, severe psychomotor impairments, urogenital abnormalities, and patterns of characteristic facial dysmorphism (9). The ATRX protein exists as two isoforms (180 and 280 kDa) and is highly enriched at GC-rich and repetitive sequences (10, 11). The C-terminus of the ATRX protein harbors the helicase/ATPase domain, classifying ATRX as part of the SNF2 (SWI/SNF2) family of chromatin-remodeling proteins (12, 13). At the N-terminus of the ATRX protein lies the ATRX–DNMT3–DNMT3L (ADD) domain, receiving its name from having cysteine-rich motifs with similar features to the DNMT3 proteins involved in DNA methylation (14, 15). The ADD domain comprises a GATA-like zinc finger, a plant homeodomain (PHD)-like zinc finger, and a C-terminal α-helix (15, 16). The presence of GATA-like zinc finger suggests a DNA/chromatin-binding role for ATRX (15), whereas the PHD-like zinc finger implies a function in chromatin regulation/transcription (14, 17). Further support of ATRX’s role in mediating chromatin remodeling and potential link with DNA methylation and gene expression has been elucidated by Gibbons and colleagues (18) who have found varied DNA methylation patterns on ATRX syndrome patients on repetitive sequences including rDNA arrays, Y-specific repeat DYZ2, and in a family of subtelomeric repeats (TelBam3.4).

Further confirming the function of ATRX as a regulator of chromatin remodeling and transcription is evidence of the formation of an ATP-dependent complex with transcription cofactor DAXX. In addition, ATRX also alters the DNase I digestion pattern and triple helix displacement activity (19). ATRX has been found to localize at pericentromeric heterochromatin (PCH), ribosomal DNA arrays on acrocentric chromosomes, telomeres, and promyelocytic leukemia (PML) nuclear bodies within mouse and human cells (13, 19, 20). Therefore, ATRX likely plays a key role in gene expression regulation. The amphipathic α-helices located on the N-terminal of DAXX can bind to one of the two regions on ATRX; the first region, located on amino acids 1,189–1,326, binds to DAXX more strongly than the second region between amino acids 321–865 (13). Although DAXX has no effect on the ATPase activity of ATRX, DAXX does attenuate its transcription repression activity and plays a role in recruiting ATRX to PML nuclear bodies (13). PML nuclear bodies have been implicated as tumor suppressors, possessing antiviral functions, and possible regulators of DNA replication and transcription (21, 22).

### Interactions with Histone Variants

The ATRX–DAXX complex plays a key role in maintaining genomic stability through its deposition of H3.3 at telomeres and PCH; this function is independent of the H3.3 deposition at regulatory elements and the histone cell cycle regulator (HIRA) complex-mediated H3.3 deposition at euchromatic regions (16, 23–25). Rather, the ADD domain of ATRX interacts with the N-terminal tail of H3.3 through its two binding pockets that are sensitive to the methylation states of specific lysine (lys) residues located on H3 tails (16, 25). One binding pocket is sensitive to unmodified Lys4 (H3K4me0) and the other is responsive to di-/tri-methylated Lys 9 (H3K9me3) (25); this readout of histone H3 modifications and interactions with heterochromatin protein 1 and MeCP2 protein allows ATRX to be recruited to heterochromatin for H3.3 deposition (16, 25, 26). In addition, ATRX conserves genomic stability by aiding in the formation of heterochromatin at intracisternal-A particle retrotransposons (27).

Within rodents, ATRX prevents transcriptional activation and accessibility to DNA-damaging elements. Although DAXX is necessary for this function, this is not linked to H3.3 deposition (27). Within mouse embryonic stem (mES) cells, the ATRX/DAXX complex protects repetitive sequences during DNA hypomethylation and imprinted loci from aberrant transcription and recombination through silencing achieved by H3K9 trimethylation; this serves to conserve genomic integrity (28, 29). In another study, it was found that the sensitivity of ATRX to histone modification H3K9 trimethylation and serine 10 phosphorylation in postmitotic neurons allowed ATRX to maintain the transcription of heterochromatin/silenced repetitive sequences during increased activity, suggesting that irregular repetitive element transcription may occur in the absence of ATRX (30). Additional evidence suggests that H3.3 is not the only histone that ATRX interacts with as parts of its regular function.

Ratnakumar and colleagues (31) observed that ATRX associates with another histone variant, macroH2A1, independent of its interaction with DAXX and H3.3. ATRX acts as a negative regulator of macroH2A1 deposition at telomeres and α-globin cluster and dysfunctional regulation is linked to the α-thalassemia phenotype in ATRX syndrome patients (31). As well, ATRX controls the binding of macroH2A1 with poly (ADP-ribose) polymerase tankyrase 1 and ATRX loss has been shown to prevent tankyrase 1 from resolving telomere sister cohesion in alternative lengthening telomere (ALT) cells (32). This cohesion is a favorable factor in tumors presenting ALT that overcome the gradual loss of telomeric DNA after each cell division; the cohesion promotes recombination between sister telomeres, which is crucial for DNA repair and telomere maintenance, and restricts recombination between non-sisters that would affect cell growth (32). These studies highlight ATRX’s role in maintaining the integrity of the genome through modifications of heterochromatin.

### Cell Cycle Regulation

Aside from histone deposition, ATRX also partakes the responsibility of activities regulating cell cycle and maintaining the stability of the genome. ATRX depletion within HeLa cells has been shown to induce lobulated nuclei and intranuclear bridges during interphase, poor cell proliferation and viability, lengthened transition between pro-metaphase and metaphase, abnormal chromosome congression, and reduced sister chromatid cohesion (10). ATRX absence has been strongly linked to DNA damage and replicative stress (33, 34). Conte and colleagues (35) discovered that multiple cell types of ATRX-null mice are sensitized to agents that cause DNA damage and apoptosis through intrinsic pathways dependent on the DNA damage checkpoint p53, suggesting that ATRX normally inhibits p53-mediated apoptosis. In another *in vivo* study, rodent GBM models exhibiting ATRX and p53 loss demonstrated an impairment in non-homologous end joining (NHEJ) DNA repair, had decreased amounts of NHEJ-related proteins pDNA-PKcs, and were more responsive to double-stranded DNA-damaging therapy than controls with p53 loss alone but ATRX maintained (33). *In vitro* analysis of mES and human cell lines revealed that ATRX associates at DNA damage sites and interacts with the Mre11–Rad50–Nbs1 protein complex, which is involved in repairing double-strand breaks and restarting stalled replication forks (34, 36). Watson and authors (37) observed that ATRX depletion within mouse neuroprogenitor cells (NPCs) had augmented replicative stress-induced DNA damage that was amplified by p53 loss at PCH and telomeres, as well as enhanced telomeric defects including telomeric fusions. Moreover, they identified that ATRX-null NPCs were sensitive to DNA damage caused by secondary DNA structure G4-stabilizing ligand telomestatin, which suggests a role in G4 replication for ATRX (37). Through work on ATRX-null mouse embryos, Seah and colleagues (38) demonstrated an absence of dentate granule cells within the neocortex and hippocampus; this was attributed to an increased rate of p53-mediated apoptosis in the hippocampus and the basal telencephalon. This was accompanied by an increased expression of *Cyclin G1* and *p21*, both of which are known targets of p53. Finally, increased apoptotic death rate was not observed in p53 mutant ATRX-null mice (38). These studies present a critical function for ATRX for regulating cell cycle-related activities and preserving the stability of the genome.

## Implications of ATRX Mutation in ATRX Syndrome

Mutations linked to the ATRX syndrome mostly cluster within the helicase and PHD domains of ATRX (39). Although mutations within both domains present similar clinical phenotypes and although there seems to exist a clinical spectrum and severity of symptoms, including MR, gross motor ability, genital abnormality, and α-thalassemia, studies have correlated high psychomotor impairments to mutations within the PHD domain (39, 40). However, there still remains uncertainty in regard to whether mutations in specific domains define symptoms as one study has associated severity of urogenital abnormality with mutations within the PHD domain (39), whereas another, based on a larger cohort of patients, has linked it to mutations within the C-terminus (9). Even within related individuals with the same mutation, severity of MR can differ (41). Patients with ATRX syndrome have also presented white matter abnormalities, delayed myelination, and non-specific brain atrophy (42, 43). In fact, ATRX-null mice express significant neuronal loss within the neocortex and hippocampus and decreased forebrain size due to heightened apoptosis during early stages of corticogenesis; this implies that ATRX may be required for cell survival during early (E11-13.5) and postnatal neuronal differentiation and may be linked to the MR seen in human patients (44). Future studies will shed more light on the pathogenesis of the ATRX syndrome.

## ATRX in Gliomas

Although significant advances have been made in the molecular aspects of brain tumors, deciphering a comprehensive role for ATRX in gliomas is still in its infancy. So far, studies have found a strong association of IDH canonical mutations and ATRX mutation (45, 46), whereas cooccurrence of 1p/19q codeletion and ATRX loss have been nearly non-existent; enabling neuropathologists to be able to make determine whether a tumor is of astrocytic or oligodendrocytic lineage without requiring both studies (8, 45). ATRX inactivation within gliomas can be due to mutations, deletions, gene fusions, or an amalgam of these causes (8). Ikemura and colleagues have demonstrated the feasibility of detecting ATRX protein expression using immunohistochemistry and correlating these expression levels with mutation status (47); this significantly simplifies the incorporation of ATRX status detection in clinical practice, given that standard sequencing methods would be difficult to apply for a large gene such as ATRX. Furthermore, ATRX mutations correlate with other prominent features including the ALT phenotype, TP53 mutations, and occur most often in astrocytic tumors (45, 48, 49). Platelet-derived growth factor receptor alpha gene (PDGFRA) amplification has also been shown to be significantly associated with ATRX loss and the ALT phenotype; future studies should look in whether possible inhibition of the PDGFRA signaling cascade may serve as a specialized therapeutic intervention within these subset of glioma patients (49, 50). Interestingly, Kannan and colleagues (45) reported that within their cohort, mutations related to ATRX cofactor DAXX were not found in LGGs and therefore in these tumors, interactions with histone is not as important perhaps. In terms of prognostication, low-grade glioma patients with ATRX retention and IDH mutations have lower progression-free survival and overall survival (OS) than tumors with 1p/19q codeletion and IDH mutations and longer time to treatment failure than those patients with IDH mutation and wild-type ATRX (55.6 vs. 31.8 months, respectively) (46, 51). This disparity aligns well with the astrocytic vs. oligodendrocytic lineage of these tumors.

In glioblastomas of young adults and pediatric patients, some studies have identified a small percentage of patients who are IDH wild-type and have a loss of ATRX expression (52–54). Ebrahimi and colleagues found these patients to have H3F3A G34 or K27 mutations, which is concordant with Ikemura and colleagues’ finding of ATRX-loss glioblastomas in younger patients being most commonly non-hemispheric in location (47, 52). On the other end of the age spectrum, the authors also identified loss of ATRX in 26 patients above the age of 55 and in 22 of these an IDH1 mutation was identified. Furthermore, contrary to some other studies, the authors showed that in IDH-mutated tumors ATRX retention is not mutually exclusive of 1p/19q codeletion (55). Together, these findings culminated in the authors recommending assessment of both IDH and ATRX status and sequencing for both IDH1/2 and H3F3A for any age group when a tumor is found to have ATRX loss and lacking IDH1/2 mutations by immunohistochemistry.

In regard to glioblastomas, reports of ATRX deficiency seem to vary; Liu and colleagues (56) observed absence of ATRX within secondary glioblastomas and more in younger patients, whereas Cai and colleagues (57) have observed lower ATRX expression more prominently in primary GBM and anaplastic gliomas than grade II gliomas and have suggested it as a malignancy marker. Therefore, it appears that additional mechanisms of tumorigenesis are involved in higher-grade gliomas and the role of ATRX is not specific in this category of tumors.

Furthermore, DNA methylation and genetic expression profiles have been found to differ among tumors with high- and low-ATRX mRNA expression; the low-ATRX subgroup had augmented methylation levels at chromatin ends (57). Cai and colleagues (57) also found that tumors with low-ATRX expression levels overexpressed genes involved in the transport, modification, and ubiquitination of proteins, in addition to (signal transduction, including GTP-related signal transduction and positive regulation of GTPase). In these tumors, transcription regulation and chromatin modification were downregulated. In addition, *in vitro analysis* of ATRX knockdown in glioma cells inhibited cell migration, increased cell death, and reduced cell viability (57). Overall, these studies highlight some important characteristics of ATRX mutations within gliomas that will aid in their detection. However, most of these studies are correlation-based and have not addressed the functionality of ATRX and its impacts when it is lost within gliomas; future studies should tailor their experiments to address the role of ATRX in the pathogenesis of gliomas so a better understanding can be acquired to implement novel, specialized treatments.

## Possible Therapeutic Interventions for ATRX-Deficient Gliomas

A substantial contribution of the inclusion of molecular parameters in glioma diagnosis has been the refinement of diagnostic and prognostic regimens. Numerous studies have focused their research on the ALT phenotype that allows cancer cells to escape replicative senescence and has definitive features including the presence of extrachromosomal C-circles, PML nuclear bodies (APBs), and telomeric sister chromatid exchange (TSCE) (55, 56). As discussed earlier, ATRX plays a pivotal role in the interaction of macroH2A1 and tankyrase (32) and highlights the ATRX–macroH2A1–tankyrase axis as a potential therapeutic target within ALT-positive, ATRX-mutant/loss tumors. Furthermore, Ramamoorthy and Smith (32) found forced sister cohesion promotes TSCE and that tankyrase overexpression resulted in the resolution of telomere cohesion, decreased recombination events, and increased copying of non-homologous telomeres, thereby impeding cell growth. Forced resolution of sister telomere cohesion was recommended as a plausible treatment target for ALT-positive tumors within ATRX-mutant/loss gliomas; identifying small peptides/molecules that can bind to macroH2A1 to release tankyrase or interrupting the interaction between macroH2A1 and PARsylated tankyrase 1 through PAR-binding domain of macroH2A1 was a goal the authors suggested for new research (32). Another study found that ectopic ATRX expression within telomerase-deficient, ALT-positive osteosarcoma epithelial (U-2 OS) cells led to DAXX-dependent reduction of several features of the ALT phenotype (58), signifying that ATRX loss is imperative for the maintenance of the ALT phenotype (58). Clynes and colleagues’ (58) study highlights that another route that can be taken toward targeting the ALT pathway may be to reduce replicative stress, possibly through nucleoside supplementation. However, ATRX deficiency alone may not trigger the ALT phenotype (59), therefore, caution should be exercised with implementing treatment strategies targeting ATRX-mutant/loss tumors.

Flynn and authors (59) observed aberrant levels of telomeric repeat-containing RNA and prolonged association of replication protein A (RPA) with telomeric ssDNA in ATRX-mutant/loss, ALT-positive cells, whereas under normal circumstances this DNA replication intermediate is released from the telomeres during the S phase. With the inhibition of DNA damage response (DDR) kinase ATR through VE-821, a regulator of the recombination carried out by RPA, chromosome destabilization, and cell death occurred in ALT-positive cancer cells; it should be noted, however, that ATRX knockdown did not induce cells to be hypersensitive to the serine/threonine protein kinase ATR treatment (59). In contrast, Deeg and colleagues (60) have reported that ATR inhibition alone is not adequate to treat ALT-positive tumors; sensitivity to ATR inhibition did not rely on the presence of ALT activity but rather was dependent on specific cell line lineage and other factors. Interestingly, another study has found ATM- (involved in DDR as like ATR) or p53- (effector protein of the ATM pathway) deficient cancer cells are more responsive to ATR inhibition treatment and result in apoptosis than normal cells (61). This potency of reduced cell survival was increased in most cancer cell lines when the ATR inhibitor VE-821 was paired with genotoxic agents, especially cisplatin; a significant synergy between VE-821 and cisplatin was also seen within ATM-/p53-deficient cells (61). The authors suggested ATR inhibition coupled with cisplatin as a treatment option for p53-deficient tumors that increase their cell survival by reducing DNA damage through ATR (61). Therefore, ATR inhibition may serve as a plausible solution to control tumor progression in ATRX-deficient tumors displaying p53 loss as well.

## Future Directions

While the identification of the role of ATRX in normal cell function and tumorigenesis has been a key step forward, additional research is warranted.

### Diagnostic Challenges

#### Optimal Tissue Collection, Storage, and Analysis

Practical hurdles are foreseeable with the use of molecular diagnostics. Although with its updated classification of CNS tumors WHO has tried to implement a consensus on diagnostic practices worldwide, there still remains a lack of guidelines on which molecular techniques to use (62). With the addition of many clinically relevant biomarkers, a major challenge is the limited amount of tissue available for molecular tests; therefore, there lies a need for the advancement of technology that can perform multiplexed/global profiling for simultaneous tests of various biomarkers (63). Formalin-fixed paraffin-embedded tissue (FFPE) remains as a popular method of tissue processing; however, biosample processing also needs to be updated to adhere for future applications of molecular diagnostics as currently FFPE degrades proteins for standard analysis (63). A survey among members of the European Confederation of Neuropathological Societies revealed heterogeneity within and between countries with regard to access to molecular diagnostics, molecular techniques, and laboratory practices, with low-income countries reporting to use fewer biomarkers in diagnostic tests (62).

#### Logistic Barriers

The newly proposed integrative approach includes other technical challenges as well, including training laboratory personnel with molecular testing, acquiring skills to interpret the combined information from traditional tests and molecular pathology, assuring reliability, reproducibility, and quality control of molecular testing, treatment difficulties with those patients who have been diagnosed with uncertain NOS (not otherwise specified) gliomas and increased costs for the new generation of technology (64, 65). Hence, huge changes to existing infrastructures and resources are foreseeable to bring about equal access to molecular diagnostics worldwide, which includes creating new regulating and accreditation agencies to ensure quality and competence of laboratories, setting up standardized procedures, references, and testing software, generation of health-care schemes and insurance policies for equal coverage of these tests, determining ethical bindings and a need for higher understanding of pathology informatics so that the multitude of data generated from molecular tests can be transformed into pathology reports relevant for clinical needs (62, 65, 66).

### Opportunities in Future Clinical Trials

As part of the spectrum of therapeutic opportunities, controlling PDGFRA amplification may be another route that can be explored in managing tumors with ATRX loss (49, 50) as well elucidating the connection between ALT and ATRX loss. However, understanding the pathogenesis of gliomas lacking ATRX is more crucial before specific treatment regimens can be developed; there still exists a large knowledge gap in how these subset of gliomas behave differently from the others and what underlies their tumor progression.

Traditional approaches toward diffuse LGGs has been the “wait-and-see” method (63). A recent survey among Canadian neurologists also demonstrated a high variability in the clinical management of LGGs (67). The ability to categorize LGG patients into molecular-based, prognostically relevant subgroups can help refine clinical practice and trial design (63). This presents an opportunity for further exploration.

## Author Contributions

PN and AM performed literature review and analysis and were involved in the writing of the manuscript. SD performed critical analysis and editing of the manuscript.

## Conflict of Interest Statement

The authors declare that the research was conducted in the absence of any commercial or financial relationships that could be construed as a potential conflict of interest.
